# Start-Up of an Anaerobic Dynamic Membrane Digester for Waste Activated Sludge Digestion: Temporal Variations in Microbial Communities

**DOI:** 10.1371/journal.pone.0093710

**Published:** 2014-04-02

**Authors:** Hongguang Yu, Qiaoying Wang, Zhiwei Wang, Erkan Sahinkaya, Yongli Li, Jinxing Ma, Zhichao Wu

**Affiliations:** 1 State Key Laboratory of Pollution Control and Resource Reuse, School of Environmental Science and Engineering, Tongji University, Shanghai, PR China; 2 Istanbul Medeniyet University, Bioengineering Department, Kadıköy, Istanbul, Turkey; 3 Laboratory of Polymères, Biopolymères and Surfaces, UMR 6270, University of Rouen-CNRS-INSA, Boulevard Maurice de Broglie, Mont-Saint-Aignan, France; Oak Ridge National Laboratory, United States of America

## Abstract

An anaerobic dynamic membrane digester (ADMD) was developed to digest waste sludge, and pyrosequencing was used to analyze the variations of the bacterial and archaeal communities during the start-up. Results showed that bacterial community richness decreased and then increased over time, while bacterial diversity remained almost the same during the start-up. *Proteobacteria* and *Bacteroidetes* were the major phyla. At the class level, *Betaproteobacteria* was the most abundant at the end of start-up, followed by *Sphingobacteria*. In the archaeal community, richness and diversity peaked at the end of the start-up stage. Principle component and cluster analyses demonstrated that archaeal consortia experienced a distinct shift and became stable after day 38. *Methanomicrobiales* and *Methanosarcinales* were the two predominant orders. Further investigations indicated that *Methanolinea* and *Methanosaeta* were responsible for methane production in the ADMD system. Hydrogenotrophic pathways might prevail over acetoclastic means for methanogenesis during the start-up, supported by specific methanogenic activity tests.

## Introduction

With the widespread applications of biological processes for wastewater treatment, large quantities of waste activated sludge (WAS) are generated along with the degradation of organic pollutants. The disposal and utilization of WAS have drawn considerable attention worldwide [1]. Anaerobic digestion (AD) is one of the most widely used process because of its ability to produce biogas, reduce the amount of sludge, destroy pathogens, and limit the production of odor [2]. In traditional AD, WAS is usually thickened to reduce its volume before further processing. This requires thickening equipment. Hydraulic retention time (HRT) and solid retention time (SRT) are identical, which prolongs HRT and reduces the flexibility of the operation [1], [2]. Anaerobic membrane digesters (AMDs), which integrate anaerobic digesters with anaerobic membrane bioreactors (AnMBRs), have several advantages over conventional AD. For example, AMD can provide a co-thickening potential, in which sludge thickening and digestion can be performed simultaneously [3]. AMD processes can also decouple HRT from SRT, which means that WAS can be treated with smaller footprint and higher organic loading rates.

In recent years, microfiltration (MF) and ultrafiltration (UF) membranes have been used in AMD processes [3], [4]. However, specific obstacles, such as the high cost of membrane modules and low membrane flux, still hinder the practical applications of AMDs [5]. In this respect, dynamic membrane technology might be a promising solution. Dynamic membranes can be formed and re-formed *in situ*, and the dynamic layer can be replaced by a newly deposited layer in case of membrane fouling. This cuts down the expense of purchasing and physically replacing new membranes [6]. High filtration flux can be achieved in anaerobic dynamic membrane reactors (AnDMBRs) [7]. To date, most related studies have focused on AnDMBR processes for municipal wastewater treatment [7], [8]. However, the information on anaerobic dynamic membrane digesters (ADMDs) in the context of sludge digestion has been very limited. The use of ADMDs for WAS digestion is worth investigating due to the prominent advantages of the process over traditional AD processes.

In anaerobic WAS digesters, microorganisms play an important role in the process, and a variety of bacteria and archaea are involved in the metabolism. Recently, molecular biology approaches, such as polymerase chain reaction (PCR)-denaturing gradient gel electrophoresis (DGGE) and 16sRNA clone libraries, have been widely used in exploration of the microbial community in AD or AnMBR systems [9]–[11]. However, low sequencing depth of those methods, which represents a mere snapshot of the dominant members and hinders the comprehensive characterization of the microbial community structure [12]. Pyrosequencing developed by Roche 454 Life Science (Brandford, CT, U.S.) is a high-throughput analytical approach that can provide enough sequencing depth to cover the complex microbial communities [13], [14]. It has been used in investigating microbial diversity in conventional AD and AnDMBR processes [15]–[18]. However, because of the use of dynamic membranes, the microbial composition and structure of ADMD might differ considerably from those of conventional AD and AnMBR within the context of wastewater treatment. For these reasons, it is essential to investigate the microbial composition and dynamics in a WAS-receiving ADMD. Pyrosequencing can provide comprehensive insights into the dynamics of the microbial communities during the start-up period of the ADMD system.

The overarching goal of this study is to characterize the temporal changes of bacterial and archaeal communities during the start-up of ADMD process for WAS digestion. By using 454 high-throughput pyrosequencing, the community structures and compositions of bacteria and archaea were investigated over time during the start-up stage in order to facilitate understanding the ADMD process.

## Materials and Methods

### Ethics statement

No specific permissions were required for these locations/activities of our field experiments. The field studies also did not involve endangered or protected species.

### Experimental setup and operation

The experimental setup used in this study is shown in Figure S1. The ADMD system consisted of a completely mixed anaerobic digester (effective volume of 67 L) coupled with a submerged anaerobic dynamic membrane reactor (effective volume of 2 L). This facilitated convenient membrane cleaning and replacement in the membrane zone while maintaining the main digester strictly anaerobic at all times. The influent flow rate was about 17 L/d. The HRT and SRT of ADMD were 6 d and 20 d, respectively. A flat-sheet dynamic membrane module was mounted in the membrane zone, which was made of Dacron mesh (pore size  = 39 μm). The surface area of dynamic membrane module was 0.038 m^2^. A peristaltic pump was installed to recycle sludge from the anaerobic digester to the dynamic membrane zone at a recirculation ratio of 300%, and another peristaltic pump was used to withdraw permeate from the dynamic membrane module. The effluent flow rate and trans-membrane pressure (TMP) were monitored using a flowmeter and a pressure gauge, respectively. The membrane module was operated with an intermittent suction mode (10-min filtration and 2-min pause) with an instant flux of about 15 L/(m^2^·h). Biogas production was measured according to the volume of biogas collected in the wetted gas collector (LMF-1, Duoyuan Instrument Technology Co., Ltd., China), in which the gas pressure was maintained at a pressure of 1 atm. The liquor level in the system was controlled using an elevated influent tank. Electric heaters controlled by temperature sensors were used to maintain the temperature of the system at 35±2 °C. Biogas was recycled using a diaphragm gas pump (KNF, Germany) on an intermittent working mode (1-min on and 20-min off) to scour membrane surfaces for fouling control, and the specific gas demand (SGD) per unit projected area of the riser zone were controlled at 25.0 m^3^/(m^2^·h) during biogas scouring. Physical cleaning was conducted for the dynamic membrane when TMP increased to 30 kPa.

**Figure 1:**
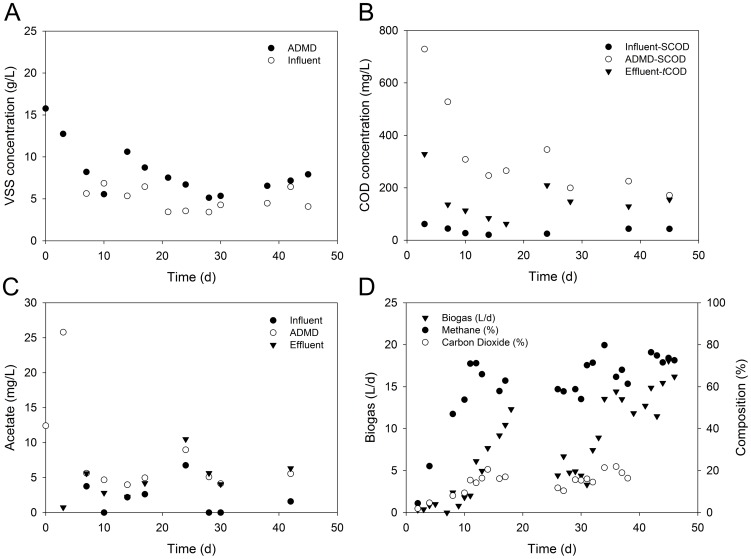
WAS digestion during the start-up stage. (A) VSS; (B) SCOD; (C) acetate; (D) biogas production and composition.

In the experiment, excess sludge from the Quyang wastewater treatment plant (WWTP) (Shanghai, China) (31°16′45″ N 121°29′08″ E) was used as the influent of the ADMD system after passing through a mesh (pore size  = 0.9 mm). The characteristics of the influent WAS are shown in Figure 1. Inoculum was collected from an anaerobic digester of the Bailonggang WWTP in Shanghai, China (31°14′42″ N 121°43′51″ E). The main features of the inoculum are as follows: pH 7.18±0.04, total suspended solids (TSS) 41.69±0.08 g/L, volatile suspended solids (VSS) 21.41±0.30 g/L, total chemical oxygen demand (*t*COD) 33111±363 mg/L, soluble chemical oxygen demand (SCOD) 1255±15 mg/L. The ratio of inoculum to substrate (v/v) was 1∶1.

### Microbial diversity analysis

#### DNA extraction and PCR amplification

For a full understanding of the microbial community dynamics during the start-up of ADMD system, sludge samples were collected on days 0, 7, 14, 28, 38, and 46, and stored at −20°C before further analysis.

Microbial DNA was extracted from sludge samples using an E.Z.N.A.^®^ Soil DNA Kit (Omega Bio-tek, Norcross, GA, U.S.) according to manufacturer's protocols. The V1–V3 and V3–V5 regions of the bacteria and archaea 16S ribosomal RNA gene were amplified by polymerase chain reaction (95 °C for 2 min, followed by 25 cycles at 95 °C for 30 s, 55 °C for 30 s, and 72 °C for 30 s and a final extension at 72 °C for 5 min) using primers 27F (5′-AGAGTTTGATCCTGGCTCAG-3′)/533R (5′-TTACCGCGGCTGCTGGCAC-3′) and Arch344F (5′-ACGGGGYGCAGCAGGCGCGA-3′)/Arch915R (5′-GTGCTCCCCCGCCAATTCCT-3′), respectively. PCR reactions were performed in a 20 μL mixture containing 4 μL of 5× FastPfu Buffer, 2 μL of 2.5 mM dNTPs, 0.8 μL of each primer (5 μM), 0.4 μL of FastPfu Polymerase, and 10 ng of template DNA.

#### 454 pyrosequencing

After purification using the AxyPrep DNA Gel Extraction Kit (Axygen Biosciences, Union City, CA, U.S.) and quantification using QuantiFluor™ -ST (Promega, U.S.), a mixture of amplicons was used for pyrosequencing on a Roche 454 GS FLX+ Titanium platform (Roche 454 Life Sciences, Branford, CT, U.S.) according to standard protocols [14]. The raw reads were deposited into the NCBI Sequence Read Archive (SRA) database (Accession Number: SRA082624).

#### Processing of pyrosequencing data

In total, 79085 and 75993 valid sequences were obtained from all 6 samples, with an average of 13181 and 12666 sequences per sample for bacteria and archaea, respectively. The resulting sequences were processed using Seqcln (http://sourceforge.net/projects/seqclean/) and Mothur (version: 1.28.0) [19]. After removing low quality sequences (quality score <25) and sequences shorter than 200 bp, with homopolymers longer than six nucleotides, and containing ambiguous base calls or incorrect primer sequences, a total of 56926 and 60634 high-quality sequences were produced with an average length of 474 and 508 bp per sequence for bacteria and archaea, respectively. Sequences were aligned against the silva database (version: SSU111 http://www.arb-silva.de/) using k-mer searching (http://www.mothur.org/wiki/Align.seqs). Potentially chimeric sequences were detected using UCHIME (http://drive5.com/uchime) and removed. The remaining reads were pre-clustered (http://www.mothur.org/wiki/Pre.cluster) and then clustered using uncorrected pairwise algorithm. In addition, operational taxonomic units (OTUs) were defined as sharing more than 97% sequence identity using Furthest neighbor method (http://www.mothur.org/wiki/Cluster). The total number of OTUs at 97% similarity level was 16663 and 5684 with an average of 2777 and 947 OTUs per sample for bacteria and archaea, respectively. Rarefaction curves, Chao1 richness estimator (http://www.mothur.org/wiki/Chao), the Shannon index (http://www.mothur.org/wiki/Shannon), and the Good's coverage (http://www.mothur.org/wiki/Coverage) were created using Mothur (version: 1.28.0) for each sample [19]. Taxonomic classification down to the phylum, class, order, family, and genus level was performed using Mothur (http://www.mothur.org/wiki/Classify.seqs) via the aforementioned silva database with a set confidence threshold of 80% [20]. Principle component analysis (PCA), heat map representation, and cluster analysis were performed via R Project (http://www.r-project.org/).

### Specific methanogenic activity (SMA) tests

SMA tests were performed with 134 mL glass assay bottles sealed with rubber stoppers and aluminum crimps. Two different substrates, acetate and H_2_/CO_2_, were used for specific acetoclastic methanogenic activity (SAMA) and specific hydrogenotrophic methanogenic activity (SHMA) tests, respectively. For SAMA tests, the reaction volume (25 mL) in each bottle was composed of ADMD sludge (approximately 2 g VSS/L), 10 mL basal medium, and acetate (2.5 g COD/L) [21]. The pH was adjusted to 7.0–7.5. Bottles were flushed with N_2_ gas for 5 min and then kept in a shaker (100 rpm) at 35±1 °C. The gas in the bottle headspace was analyzed periodically after overnight incubation [21]. All the analyses were performed in triplicate. This was followed by a blank control, which reproduced the test with deionized water instead of the substrate solution. For SHMA tests, the reaction volume (25 mL) in each bottle was composed of ADMD sludge (approximately 2 g VSS/L) and 10 mL basal medium [21], with pH adjusted to 7.0–7.5. Bottles were flushed with H_2_/CO_2_ (80%∶20% v/v) for 5 min and pressurized to 1 atm, and then kept in a shaker (180 rpm) [22]. The temperature was maintained at 35±1 °C. The gas in the bottle headspace was also analyzed periodically after overnight incubation [21]. All the analyses were performed in triplicate along with a blank control, which was treated with N_2_ instead of H_2_/CO_2_. Both SAMA and SHMA were calculated according to the methods reported by Yukselen [23].

### Other analytical methods

Sludge samples were centrifuged at 6000 rpm for 10 min and then filtered through normal quantitative papers. The filtrate was analyzed for volatile fatty acids (VFAs) and SCOD. The analyses of COD, TSS, and VSS were conducted in accordance with standard methods [24]. Gas composition (CH_4_ and CO_2_) was measured using a gas chromatography (6890N, Agilent, U.S.) equipped with a thermal conductivity detector (TCD). The determination of VFAs was described in a previous study [25].

## Results and Discussion

### Digestion performance

The ADMD system was operated for 46 days during the start-up stage (Figure 1). The influent VSS concentration was 4.90±1.29 g/L (n = 11), while the average VSS concentration of ADMD sludge remained about 8.06 g/L after 7 days, which was about 1.6 times of that of the influent. This indicated that the ADMD system also had thickening functions along with the digestion of WAS. A VSS reduction rate of about 48.7% was achieved during the start-up stage. The SCOD concentration of influent was 35±15 mg/L (n = 8), while SCOD of ADMD sludge and *t*COD of effluent were 315±183 mg/L (n = 10) and 152±79 mg/L (n = 9), respectively. The SCOD of ADMD sludge was about 8 times of that of influent, indicating that soluble compounds had been released into the system. This is because sludge hydrolysis can be expressed by the increase of SCOD [26]. The *t*COD of ADMD permeate was lower than the SCOD of ADMD sludge, indicating that dynamic membrane in ADMD was able to partially retain the dissolved organic matters. Acetate was the predominant VFA, accounting for 91–100% of total VFAs. Its concentration is shown in Figure 1C. The influent acetate concentration varied within a range of 0–2.1 mg/L. As for ADMD sludge, slight accumulation of acetate was observed on day 3, and the acetate concentration decreased afterwards and remained 5.4±1.6 mg/L (n = 8). Meanwhile, the acetate concentration in effluents was 5.2±2.6 mg/L (n = 8) from day 7 during the start-up, which was almost the same as that of ADMD sludge, indicating that acetate could permeate through dynamic membrane in the ADMD.

Figure 1D depicts biogas production in the ADMD system during start-up. Biogas production was converted to standard temperature and pressure (0 °C and 1 atm). Biogas production was observed on day 2 shortly after start-up, and it was found to increase in an almost linear fashion from day 2 to day 18. The deficiencies in biogas production from day 20 to day 31 were attributed to the malfunction of the gas collection system. From day 34 to day 46, biogas production remained 12.58±4.59 L/d (n = 11). The methane content of the biogas increased from day 2 to day 11, and remained 67.8±7.6% (n = 21) from day 11 to day 46. Biogas production became relatively steady at the end of the start-up stage, and the methane yield per gVSS-removed reached 0.32 L/(gVSS·d) from day 34 to day 46.

### Richness and diversity of microbial communities

In the present study, 2447–3311 and 860–1055 OTUs were clustered at a dissimilarity level of 0.03 for analyzing the bacterial and archaeal communities, respectively. The number of effective sequence reads and of OTUs were both higher than that of conventional molecular biology methods [9]–[11]. This could indicate that 454 pyrosequencing could better characterize of microbial consortia [12]. As listed in Table 1, in the bacterial domain, the number of OTUs decreased from day 0 to day 28 and increased from day 28 to day 46. Changes in Chao1 were also used to estimate the total number of OTUs. It showed a trend similar to that of OTUs, but the lowest Chao1 estimators occurred on day 38. Both indicators, OTUs and Chao1, demonstrated that the richness of the bacterial community decreased and then increased during the start-up period. These findings were also confirmed by rarefaction curves (Figure S2A). The Shannon index, which estimates the diversity of microbial population, fluctuated within the range of 6.66–7.14 from day 0 to day 46, showing no significant changes in bacterial diversity during the start-up period. Unlike in the bacterial domain, in the archaeal domain, the number of OTUs increased from day 0 to day 7, decreased from day 7 to day 14, remained relatively stable from day 14 to day 38, and increased again from day 38 to day 46. The changes in the Chao1 estimator were similar to those in OTUs. The Shannon index remained within the range of 3.85–3.96 from day 0 to day 14, and increased to 4.26–4.39 from day 28 to day 46. On day 46, OTUs, Chao1, and Shannon were the highest during the start-up period, indicating the richness and diversity of the archaeal community peaked at the end of the start-up stage. Good's coverage, which represents the probability that the next read will belong to an OTU that has already been observed, can be used to evaluate the level of information contained in microbial communities [11]. As listed in Table 1, the coverage values of bacterial and archaeal community were within the range of 0.69–0.73 and 0.93–0.95, respectively, indicating that the most common phylogenetic groups were detected in our study. Similar coverage values were also observed by other researchers, and the relatively low values of coverage for bacteria might be related to the large diversity encountered in anaerobic environment [11].

**Table 1:** Diversity statistics of bacterial and archaeal community.

	Bacterial Community				Archaeal Community			
Time (d)	OTUs^a^	Chao1^a^	Shannon^a^	Coverage^a^	OTUs^a^	Chao1^a^	Shannon^a^	Coverage^a^
0	3311	10014	7.14	0.69	971	1757	3.96	0.95
7	2778	8185	6.66	0.73	1011	1969	3.94	0.95
14	2726	8069	6.98	0.73	860	1721	3.85	0.94
28	2447	7962	6.82	0.71	888	1887	4.29	0.93
38	2551	7823	6.85	0.73	899	1821	4.26	0.94
46	2850	8983	7.03	0.70	1055	2075	4.39	0.94

a Values were defined at a dissimilarity level of 0.03.

### Bacterial consortia dynamics

**Figure 2:**
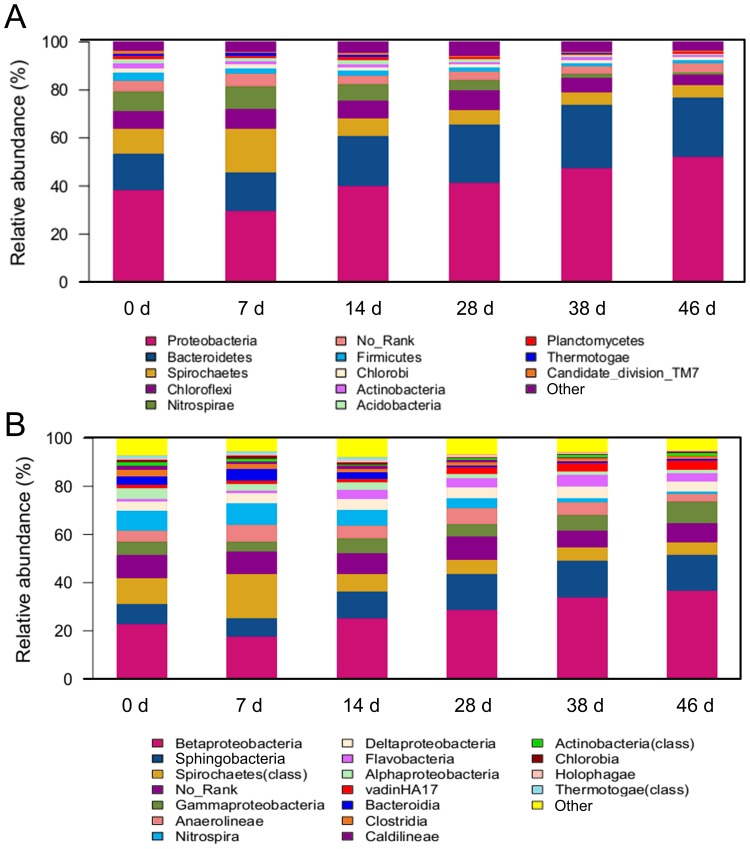
Changes in the bacterial community structure during the start-up of ADMD system. (A) phylum level; (B) class level. Relative abundance is defined as the number of sequences affiliated with that taxon divided by the total number of sequences per sample (%). Phyla or classes accounting for less than 1% of relative abundance are regarded as other.

Figure 2 illustrates the distribution of the bacterial community during the start-up of the ADMD system. As shown in Figure 2A, *Proteobacteria* and *Bacteroidetes*, whose relative abundance was within the range of 29.6–52.3% and 15.4–26.1%, respectively, were the two most predominant phyla. *Proteobacteria* and *Bacteroidetes* have been reported to be common and abundant in anaerobic sludge digesters [11], [17], [27], MBR and AnDMBR systems [18], [20]. The relative abundance of *Proteobacteria* was 38.1% on day 0, decreased to 29.6% on day 7, and increased to 52.3% on day 46. The relative abundance of *Bacteroidetes* increased to 24.2% during the beginning 38 d and remained relatively stable within the range of 24.2–26.1% from day 38 to day 46. Results showed that at the end of the ADMD system's start-up stage, *Proteobacteria* and *Bacteroidetes* accumulated and overwhelmed other phyla. They were able to degrade a wide range of macromolecules and xenobiotic compounds [28].

As shown in Figure 2B, five classes of *Proteobacteria*, i.e., *alpha*-, *beta*-, *gamma*-, *delta*-, and *epsilon*-, were observed during the start-up of ADMD, but *beta*-, *gamma*-, and *deltaproteobacteria*, which accounted for 25.6–49.4% of total reads at the class level, were the major classes of *Proteobacteria*. *Betaproteobacteria* was the most predominant class, of which the relative abundance decreased from day 0 to day 7 and increased from day 7 to day 46. Previous studies also showed that *Betaproteobacteria* was the major class of *Proteobacteria* in conventional AD, MBR and AnDMBR systems [17], [18], [20]. *Betaproteobacteria* has been reported to be the most dominant group in propionate-, butyrate-, and acetate-utilizing microbial communities in anaerobic digesters [27]. Accordingly, the high abundance of *Betaproteobacteria* might lead to VFA consumption, and low VFA concentration was observed during the start-up of the ADMD system (Figure 1C).

In the phylum of *Bacteroidetes*, the relative abundance of the *Sphingobacteria* class was 8.4–15.5% during the experiment, and *Sphingobacteria* became the most abundant class at the end of the start-up (Figure 2B). In MBR systems for wastewater treatment, *Sphingobacteria* was also found to be most dominant class of *Bacteroidetes* [20]. With the relative abundance of 3.54–5.10% from day 14 to day 46, *Flavobacteria* was the second abundant class at the end of the start-up. The *Cytophaga-Flavobacteria* cluster, which belongs to the phylum of *Bacteroidetes*, was capable of consuming chitin, N-acetylglucosamine and protein, and degrading high molecular mass fraction of dissolved organic material [29], [30]. In this way, the presence of *Flavobacteria* might enhance the removal of WAS pollutants.

It is worth noting that some minor classes, such as *Alphaproteobacteria* and *Clostridia*, were observed during the start-up of ADMD system (Figure 2B). It has been reported that the genera of *Rhodobacter* and *Clostridium*, which belong to *Alphaproteobacteria* and *Clostridia*, respectively, were able to carry out the bio-hydrogen production [31]. In this study, the presence of these bacteria might produce hydrogen during the AD processes. The produced hydrogen could be utilized by methanogens via hydrogenotrophic pathway, which will be further elaborated in the following section.

### Archaeal consortia dynamics

**Figure 3:**
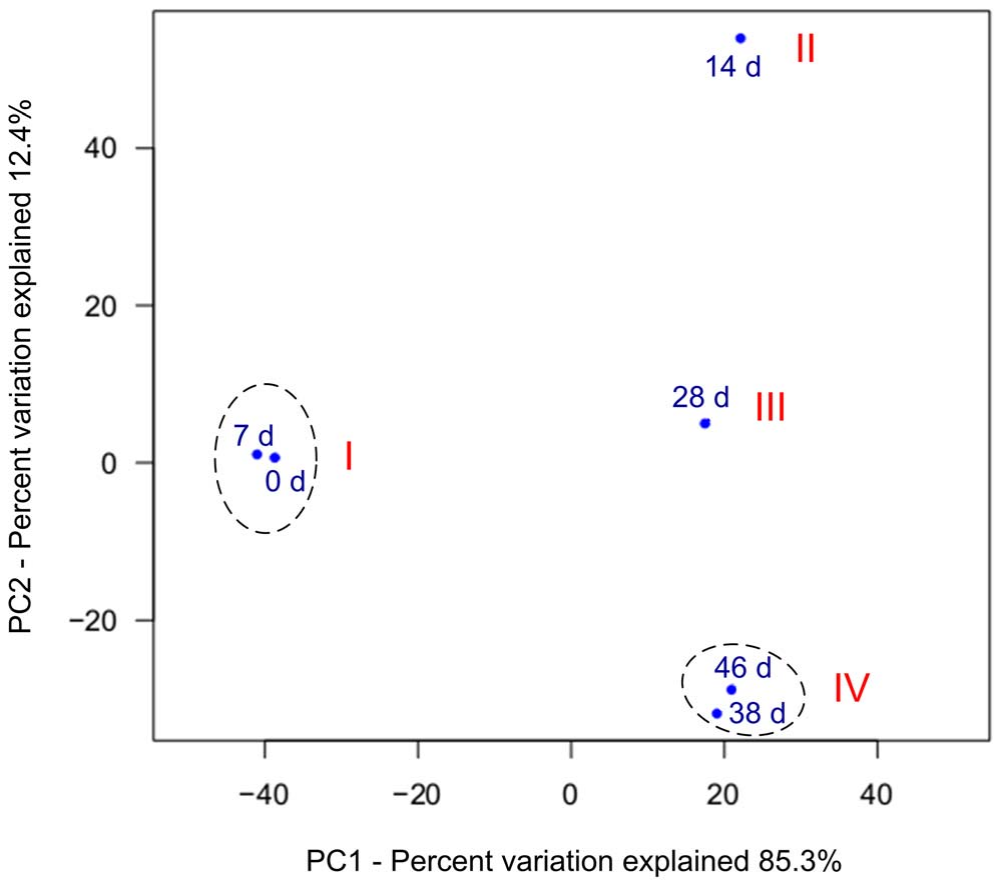
Principle component analysis (PCA) of ADMD samples taken at different times. PCA was conducted at a 3% cutoff OTU level.

**Figure 4:**
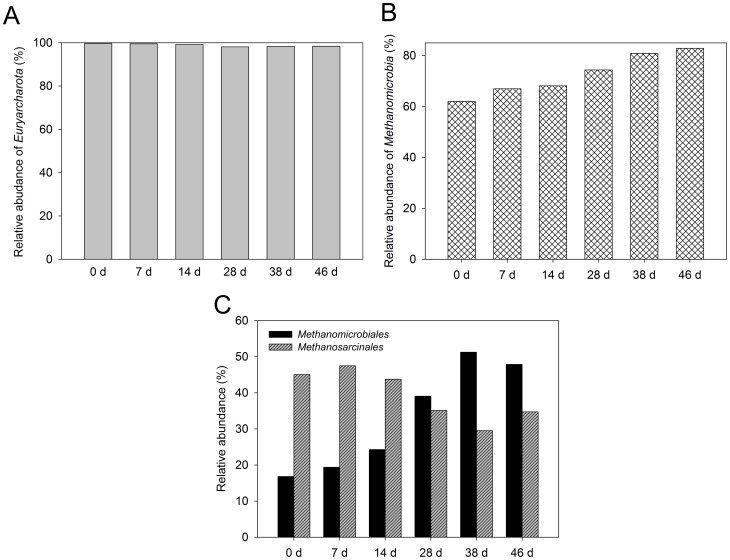
Changes in the archaeal community structure during the start-up of ADMD system. (A) phylum level; (B) class level; (C) order level. Relative abundance is defined as the number of sequences affiliated with that taxon divided by the total number of sequences per sample (%).

In order to analyze the similarity of archaeal consortia at different times, principle component analysis (PCA) was conducted using OTUs at a dissimilarity level of 0.03. As seen in Figure 3, ADMD samples were clustered into four groups: (1) Group I contains the samples on day 0 and day 7; (2) Group II contains the sample on day 14; (3) Group III is the ADMD sludge on day 28; (4) Group IV is the ADMD sludges on day 38 and day 46. Results showed that the samples on day 0 and day 7 were similar to each other. Considerable similarity was also observed between the samples taken on day 38 and those taken on day 46. In a horizontal view, the difference of Groups II, III, and IV was small, but each of them differed from Group I considerably. The above-mentioned observations indicate that a distinct shift of archaeal populations of ADMD system occurred after day 7 because PC1 was the major principle component, which accounted for 85.3% of the total variation. Archaeal consortia became relatively stable after 38 d. This was in accordance with biogas production (Figure 1D) and archaeal community structure at the order level (Figure 4C). The results indicate that the ADMD could be well started up within 38–46 d.

The changes in the archaeal consortia can be demonstrated with the variations of archaeal community structure. As shown in Figure 4A, although a small decrease in relative abundance was observed during the start-up stage, the relative abundance of *Euryarcharota* was within the range of 98.4–99.6% at the phylum level, indicating that *Euryarcharota* was the predominant phylum in the ADMD system. The relative abundance of *Methanomicrobia* increased from 62.0% to 82.9% during the start-up (Figure 4B). It was the major class in the ADMD system. At the order level (Figure 4C), *Methanomicrobiales* and *Methanosarcinales*, which together accounted for 61.9–82.6% of total reads on the order level, were the two predominant orders during the start-up stage. *Methanomicrobiales* and *Methanosarcinales* have also been found to be abundant in AnMBR system for swine manure treatment [32]. The sum of relative abundance of both orders increased over time. However, changes in individual orders were different. The relative abundance of *Methanomicrobiales* increased from day 0 to day 38 and remained relatively stable within the range of 47.9–51.3% from day 38 to day 46. However, the relative abundance of *Methanosarcinales* decreased from day 0 to day 28 and remained relatively stable within the range of 29.5–35.1% afterward. During the start-up stage, the population of *Methanomicrobiales* gradually exceeded that of *Methanosarcinales*. It became the most abundant order on day 46. It has been reported that *Methanomicrobiales* comprises hydrogenotrophic methanogens and *Methanosarcinales* contains acetoclastic methanogens [33]. The results indicated that methanogenic pathways might be altered during the start-up stage of ADMD system.

**Figure 5:**
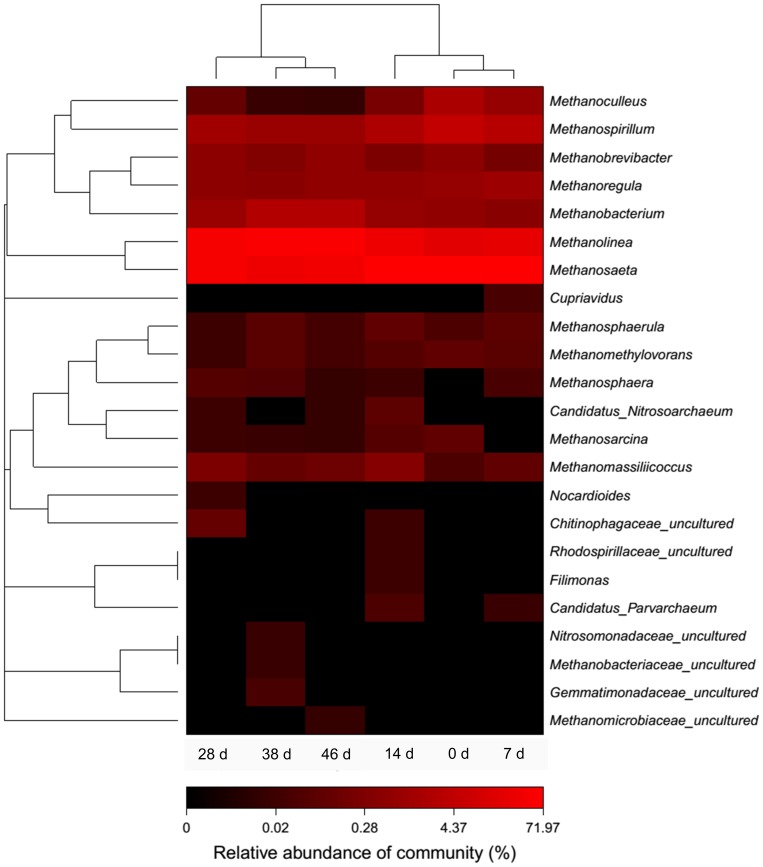
Heat map representation and cluster analysis of archaeal community at the genus level. Distance algorithm: Bray-Curtis; clustering method: complete. The color intensity of scale demonstrates the relative abundance of each genus. Relative abundance is defined as the number of sequences affiliated with that taxon divided by the total number of sequences per sample (%).

To further investigate the changes of archaeal community and functional groups, cluster analysis was conducted at the genus level. As shown in Figure 5, four clusters were identified from the six archaeal consortia using hierarchical cluster analysis: (1) Cluster I contains the ADMD sludges on day 0 and day 7; (2) Cluster II contains the ADMD sludge on day 14; (3) Cluster III is the ADMD sludge on day 28; (4) Cluster IV is the ADMD sludges on days 38 and 46. The archaeal consortia on the genus level in Cluster I (0 d and 7 d) showed considerable homology, which was also observed in Cluster IV (38 d and 46 d). These above-mentioned results are consistent with PCA at a 3% cutoff OTU level (Figure 3) and cluster analysis of bacterial community at the genus level (see Figure S3). *Methanolinea* and *Methanosaeta* exhibited considerable abundance during the start-up of ADMD system. On day 0, the relative abundance of *Methanosaeta* and *Methanolinea* were 50.0% and 10.3%, respectively. However, with the increase in abundance of the former and the decrease in abundance of the latter during the start-up, *Methanolinea* surpassed *Methanosaeta* to become the most predominant genus from day 28 to day 46. Previous studies have been done on the archaeal community in AD systems. They have reported that the acetoclastic *Methanosaeta* was the most abundant archaeal genus and the hydrogenotrophic *Methanolinea* only accounted for a small fraction of genus-assigned sequences [17]. The results of the present study were quite different, indicating that archaeal community in the ADMD system was distinct from conventional AD systems. *Methanosaeta* were found to be the dominant acetoclastic methanogens in a variety of anaerobic bioreactors when acetate concentration was low [34]. In the present study, the high abundance of *Methanosaeta* was in accordance with low acetate concentration during the start-up (Figure 1C). However, carbon dioxide contained 8–22% of biogas after 7 days (Figure 1D), which was below the range of 30–35% in AD processes [2]. These results indicated that some of the carbon dioxide might be consumed by hydrogenotrophic methanogens and used as a carbon source. This is consistent with the predominant abundance of *Methanolinea*. Based on these results, it might be speculated that *Methanolinea* and *Methanosaeta* were responsible for most of the methane production in the ADMD system. Because *Methanolinea* and *Methanosaeta* are hydrogenotrophic and acetoclastic, respectively, methanogenesis might be carried out via both hydrogenotrophic and acetoclastic pathways, but the hydrogenotrophic pathway might prevail.

**Figure 6:**
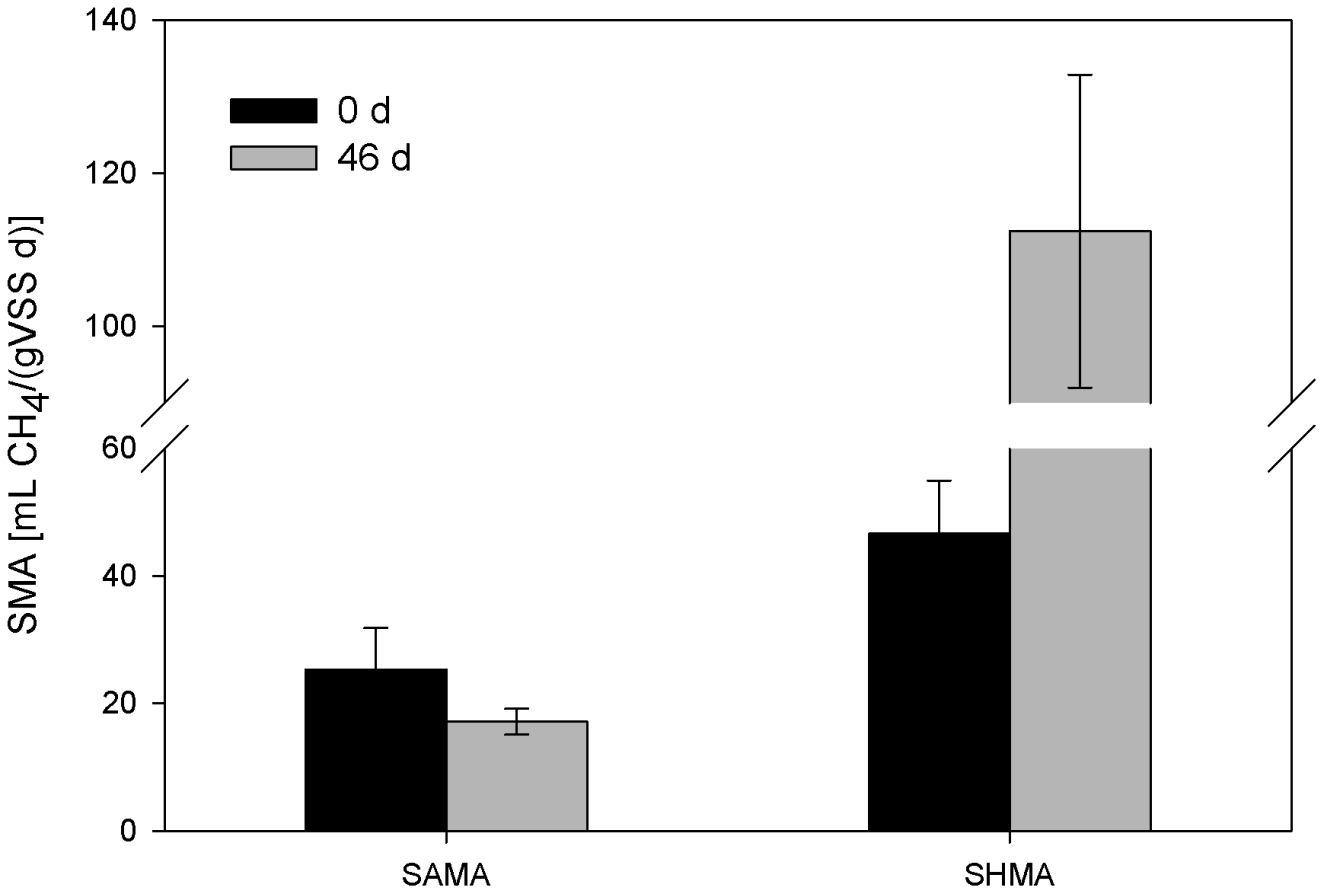
SAMA and SHMA tests of ADMD samples on day 0 and day 46. Error bars represent standard deviations of triplicate tests.

To validate the available methanogenic pathways during the start-up of the ADMD system, SMA tests were performed on day 0 and day 46 using acetate and H_2_/CO_2_ as substrates, respectively. As shown in Figure 6, SAMA on day 0 and day 46 were 25.3±6.5 mL CH_4_/(g VSS·d) and 17.1±2.0 mL CH_4_/(g VSS·d), respectively, indicating that acetoclastic methanogens became less dominant at the end of start-up. However, SHMA was 46.6±8.4 mL CH_4_/(g VSS·d) and 112.4±20.5 mL CH_4_/(g VSS·d) on day 0 and day 46, respectively, indicating that hydrogenotrophic methanogens became more dominant at the end of start-up. The results of SAMA and SHMA could not be compared directly because the concentrations of substrates were different. However, the effects of substrate concentrations could be eliminated via the comparison of SHMA/SAMA ratio. The average SHMA/SAMA on day 46 was about 3.6 times of that on day 0. The results indicated that both hydrogenotrophic and acetoclastic pathways were observed on day 0 and day 46 via SMA tests, but hydrogenotrophic pathways were more predominant at the end of the start-up of ADMD system. This is in accordance with the analysis of archaeal community structure.

## Conclusions

During the start-up, methane production improved gradually and became relatively steady in the end. Pyrosequencing showed that *Proteobacteria* and *Bacteroidetes* were the predominant phyla in bacterial consortia. In archaeal consortia, principle component and cluster analyses demonstrated that archaeal consortia experienced a distinct shift and became relatively stable after day 38, indicating that the reactor can be well started-up within 38 days. *Methanomicrobiales* gradually exceeded *Methanosarcinales*, and became the most abundant at the class level. Further analysis indicated that *Methanolinea* and *Methanosaeta* were the functional genera for methane production. Hydrogenotrophic pathways might prevail over acetoclastic pathways for methanogenesis in the process.

## Supporting Information

Figure S1 Schematic of ADMD system.
(TIF)

Figure S2 Rarefaction curves based on pyrosequencing of microbial communities. (A) bacteria; (B) archaea. The OTUs were defined by clustering sequences at a dissimilarity level of 0.03.
(TIF)

Figure S3 Heat map representation and cluster analysis of bacterial community at the genus level. The y-axis is the clustering of the 100 most abundant genera in reads. Distance algorithm: Bray-Curtis; clustering method: complete. The color intensity of scale demonstrates the relative abundance of each genus. Relative abundance is defined as the number of sequences affiliated with that taxon divided by the total number of sequences per sample (%).
(TIF)
