# Supplementary figures and images for: Start-Up of an Anaerobic Dynamic Membrane Digester for Waste Activated Sludge Digestion: Temporal Variations in Microbial Communities
Source: PLoS One. 2014 Apr 2;9(4):e93710. doi: 10.1371/journal.pone.0093710 (PMC3973557; doi:10.1371/journal.pone.0093710)

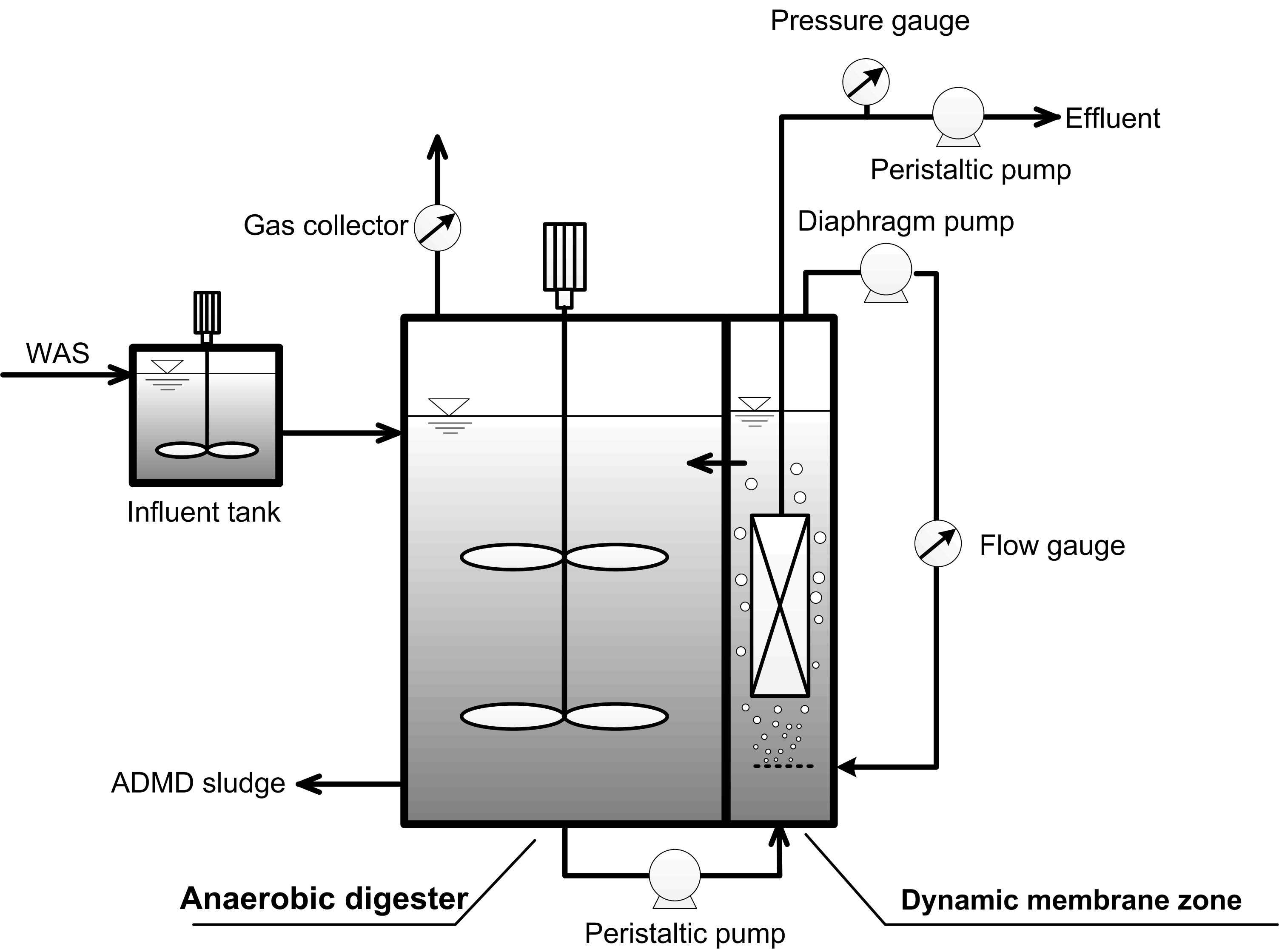

Supplement: Figure S1 — Schematic of ADMD system. (TIF) [file pone.0093710.s001.tif]
